# M1 and M2 Macrophage Polarization Correlates with Activity and Chronicity Indices in Lupus Nephritis

**DOI:** 10.3390/life15010055

**Published:** 2025-01-04

**Authors:** Chutima Chavanisakun, Rassamon Keawvichit, Nontawat Benjakul

**Affiliations:** 1 Department of Anatomical Pathology, Faculty of Medicine Vajira Hospital, Navamindradhiraj University, 681 Samsen Road, Dusit, Bangkok 10300, Thailand; 2 Vajira Pathology-Clinical-Correlation Target Research Interest Group, Faculty of Medicine Vajira Hospital, Navamindradhiraj University, 681 Samsen Road, Dusit, Bangkok 10300, Thailand; 3 Department of Clinical Pathology, Faculty of Medicine Vajira Hospital, Navamindradhiraj University, 681 Samsen Road, Dusit, Bangkok 10300, Thailand

**Keywords:** systemic lupus erythematosus, lupus nephritis, macrophages, macrophage subtypes, renal pathology

## Abstract

**Background:** Lupus nephritis (LN) is a severe manifestation of systemic lupus erythematosus (SLE), characterized by inflammation and immune dysregulation in the kidneys. The role of macrophage polarization in LN progression remains underexplored. **Objective:** This study examined the association between tubulointerstitial M1/M2 macrophage subpopulations and LN indices of activity and chronicity. **Materials and Methods:** We retrospectively reviewed 160 renal biopsy specimens in patients with LN (ISN/RPS classes II–V) from the database of the Department of Anatomical Pathology, the Faculty of Medicine Vajira Hospital, Navamindradhiraj University (2012–2021). Additional immunohistochemical analysis included CD68, iNOS, CD206, CD163, and evaluation of infiltration with M1 (iNOS+), M2a (CD206+), and M2c macrophages (CD163+). Moreover, clinical information at the time of the renal biopsy, including age, sex, and laboratory findings, was obtained from the electronic medical records. The data were correlated with the macrophage infiltration using the Spearman test. **Results:** Lupus nephritis biopsies with ISN/RPS class II–V were included (class II: 3 cases (2%), III: 30 cases (19%), III + V: 16 cases (10%), IV: 73 cases (46%), IV + V: 18 cases (11%), and V: 20 cases (12%)). In addition, the mean age of SLE patients at the time of biopsy was 33 years (range: 19–47 years). Most patients were females (n = 141; 88%). The population of CD68+ macrophages was related to serum creatinine (*p* < 0.001; rs = 0.34). We detected predominantly M2 macrophages across all LN classes, but M1 macrophages demonstrated significant correlations with the activity index (*p* < 0.001; rs = 0.43). Conversely, M2a and M2c subpopulations were strongly associated with the chronicity index (M2a: *p* < 0.001, rs = 0.48; M2c: *p* = 0.024, rs = 0.18). Total macrophages correlated with both indices (activity: *p* < 0.001, rs = 0.44; chronicity: *p* < 0.001, rs = 0.42). **Conclusions:** In lupus nephritis, the predominant population of macrophages is M2. Correlations were noted between the subpopulations of M1 and M2c macrophages and the activity and chronicity indices, respectively. In addition, macrophage populations correlated with disease progression, but the significance of this association in disease progression remains uncertain.

## 1. Introduction

Systemic lupus erythematosus (SLE) is a complex autoimmune disease that primarily affects reproductive-aged women and involves multiple organ systems. Lupus nephritis (LN), a severe manifestation of SLE, is driven by immune dysregulation and inflammation, particularly within renal tissues. LN is a significant consequence of SLE and a leading cause of morbidity and mortality in patients with SLE [1]. The prevalence of LN varies across ethnicities. A previous systematic review found a higher prevalence in Asians with more kidney involvement than that found in Caucasians [2]. Other non-neoplastic renal pathologies, such as diabetic nephropathy, in renal tumor nephrectomy and nephroureterectomy specimens in Southeast Asia are found [3]. LN affects approximately 0.1% of Asians, or roughly 4.1 cases per 100,000 individuals per year. It was observed in 53% of initially diagnosed SLE patients and 78% of SLE patients in Thailand [2,4].

Numerous studies indicate the presence of macrophages (MØ) in the pathophysiology of LN; however, the precise nature of their contribution has not yet been determined [5,6]. As essential immunocytes, macrophages mediate the initiation and progression of renal damage in numerous ways. Macrophages increase the synthesis of autoantibodies and pro-inflammatory cytokines, resulting in glomerular injury and the start of proteinuria [6,7].

Macrophages are essential in innate and adaptive immunity as well as systemic metabolism, hematopoiesis, vasculogenesis, and reproduction [8,9,10]. Macrophage differentiation refers to the process by which cells migrate to the vessel wall from peripheral blood and then into organizations, in which adhesion molecules, chemokines, and cytokines influence monocyte migration and late maturation [9], and macrophage polarization refers to the phenotypical/functional switch caused by macrophages completely differentiated in a specific tissue responding to external stimuli. Macrophages are classified into two types based on their microenvironment in various stages of lupus nephritis: traditionally activated macrophages (M1) and alternatively activated macrophages (M2) [10]. The microenvironments at different phases of LN influence the dynamic proportion between M1 and M2 macrophages in renal tissue, which determines the progression and prognosis of LN. During acute renal injury, M1 macrophages infiltrate the kidneys and release pro-inflammatory cytokines [11,12,13]. During the healing phase, however, these cells may transition to an M2 phenotype, contributing to the repair of epithelial and vascular endothelial cells and thereby causing renal fibrosis, which correlates with a worse LN outcome [14,15].

In this study, we investigated whether the presence of M1 or M2 macrophages is dependent on the index of lupus nephritis and whether there is any correlation with clinical parameters. Understanding the M1/M2 macrophage polarization axis in lupus nephritis could provide insight into disease mechanisms, aiding in the identification of potential biomarkers for disease activity and chronicity. Furthermore, this axis may serve as a therapeutic target for modulating immune responses to improve outcomes in patients with lupus nephritis.

## 2. Materials and Methods

### 2.1. Renal Tissue Specimens

In our study, we analyzed macrophage phenotypes in 160 cases of lupus nephritis patients that were classified as SLE with LN classes II to V according to the ISN-RPS LN classification [16,17]. All renal biopsy specimens included in this study were obtained prior to the initiation of immunosuppressive therapy and represented first-time biopsies for the patients. This approach ensured that the macrophage polarization and infiltration patterns observed were not influenced by prior therapeutic interventions. All diagnoses were made by nephropathologists. This study was approved by the Institutional Review Board of the Faculty of Medicine Vajira Hospital, Navamindradhiraj University (COA 190/2564).

### 2.2. Immunohistochemical Analysis

For immunohistochemistry, the following antibody reagents were used: CD68, a monoclonal mouse antibody (dilution: 1:500) (Clone PG-M1, Dako, Glostrup, Denmark); iNOS, a polyclonal rabbit antibody (dilution: 1:50) (GTX130246, GeneTex Inc., Irvine, CA, USA); CD206, a monoclonal rat antibody (dilution: 1:25) (Clone MR5D3, GeneTex Inc., North America); and CD163, a mouse monoclonal antibody (dilution: 1:200) (clone 10D6, Novocastra, Leica Biosystems Newcastle Ltd., Newcastle, UK). Whole-tissue sections were used in all cases. Blocks were sectioned with a thickness of 3 μm, using superfrost plus slides, and then the antibodies were diluted as appropriate for each type. The tissue slides were treated with bond dewax solution (Leica Microsystems) and incubated at 60 °C for 60 min. Epitope retrieval was performed by incubating the slides in bond epitope retrieval solution 1, and then they were incubated at 100 °C for 20 min. After that, the primary antibody was added, and the slides were incubated for 45 min. Then, they were rinsed with Bond wash solution (BWS) 3 times, 3% hydrogen peroxide was added, and they were incubated for 5 min and then rinsed with BWS 3 times and treated with a post-primary polymer prior to incubation for 8 min. Triple rinses were performed with BWS, and the slides were incubated with Polymer Poly-HRP IgG for 8 min. Then, they were rinsed 3 times by BWS, followed by a single rinsing with deionized water. After, the diaminobenzidine chromogen was added, and the slides were incubated at room temperature for 4 min. Then, they were triple-rinsed with deionized water prior to counterstaining the slides with hematoxylin for 5 min. Finally, the slides were dehydrated and covered with coverslips before the investigation.

### 2.3. Histopathological Evaluation

All histomorphological features in the activity index and chronicity index were re-evaluated by two nephropathologists (C.C. and N.B.) using hematoxylin and eosin (H&E)-, periodic acid–Schiff (PAS)-, and Jones’ methenamine silver (JMS)-stained tissue sections. The morphological features were scored according to the ISN/RPS classification. Interstitial fibrosis and tubular atrophy (IF/TA) were scored using the categories in the Banff allograft pathology classification [18]. The tissue preparation followed our laboratory principles [19].

### 2.4. Statistical Analysis

All statistical analyses were performed using the SPSS software version 26.0 (IBM Corp., Armonk, NY, USA). Continuous data were presented as means ± standard deviations (SDs) or medians and interquartile ranges (IQRs), depending on the distribution of the data. Categorical variables were reported as counts and percentages.

Comparisons between groups were conducted using the Kruskal–Wallis test for non-normally distributed data, followed by post hoc analysis with the Mann–Whitney U test for pairwise comparisons. The Spearman’s rank correlation coefficient (ρ) was used to evaluate correlations between macrophage subpopulations and clinical indices, including activity and chronicity scores.

*p*-values of < 0.05 were considered statistically significant. Figures and tables were generated to visualize key statistical results, including the macrophage distribution across lupus nephritis classes and correlations with clinical parameters.

## 3. Results

### 3.1. Characteristics of SLE Patients

Most LN biopsies were classified as ISN/RPS class IV (46%) and class III (19%). Patients were predominately female (88%) in this study. The mean age at the time of biopsy was approximately 33 years (Table 1). Renal function, as assessed by serum creatinine, 24 h urine protein, and dipstick urine protein, varied widely within the ISN/RPS classes. The mean serum creatinine was 1.18 mg/dL and 0.7 mg/dL before treatment and after 1-year treatment, respectively. Practically, overall, patients had severe proteinuria, indicated by dipstick urine protein levels of 4+ (47%) and 3+ (29%) (Table 1).

### 3.2. Different Tubulointerstitial Macrophage Subpopulations Among Various Lupus Nephritis Classifications

**Figure 1:**
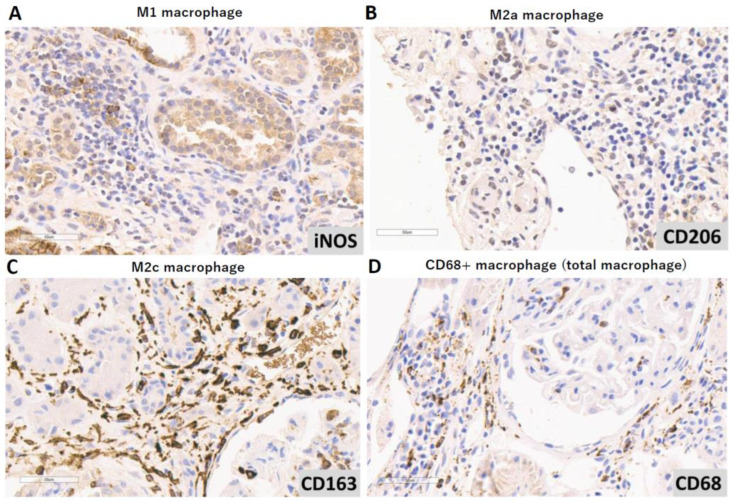
Immunohistochemical staining for subpopulations of macrophages in interstitial areas (magnification: ×40). (**A**–**D**): Antibody reagents, including anti-iNOS, anti-CD206, anti-CD163, and anti-CD68, were used to identify M1, M2a, M2c, and total macrophage cells, respectively.

Interstitial fibrosis and tubular atrophy (IF/TA) were scored using the categories in the Banff allograft pathology classification [18]. The total number of macrophages (Figure 1A; CD68+), M1 (Figure 1B; iNOS+) [19,20], M2a (Figure 1C; CD206+) [19], and M2c macrophages (Figure 1D; CD163+) [19,21] were counted in 10 HPFs in the most prominent area.

**Figure 2:**
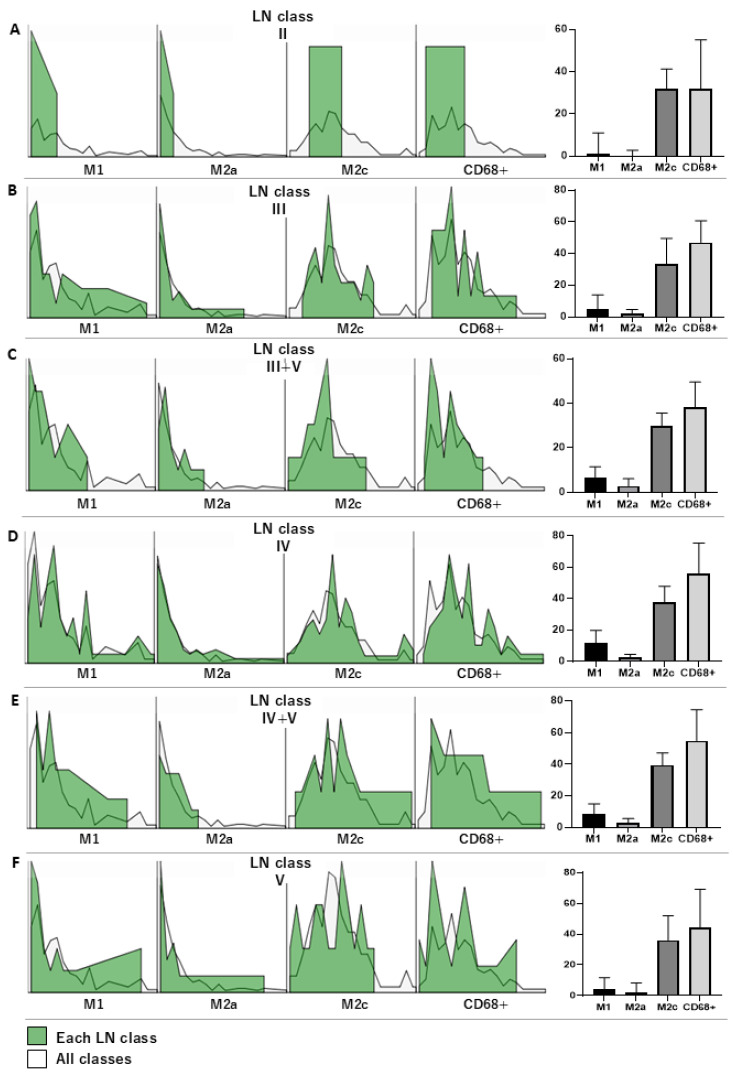
Frequency distribution of tubulointerstitial macrophage subpopulations in different LN classes. Overall histograms show frequency distributions of macrophage subpopulations in each LN class (green), including LN classes II (**A**), III (**B**), III + V (**C**), IV (**D**), IV + V (**E**), and V (**F**), compared with all LN classes (white). The median and interquartile range of each tubulointerstitial macrophage subpopulation is demonstrated concurrently in each LN class (far right figures) in their corresponding columns.

**Figure 3:**
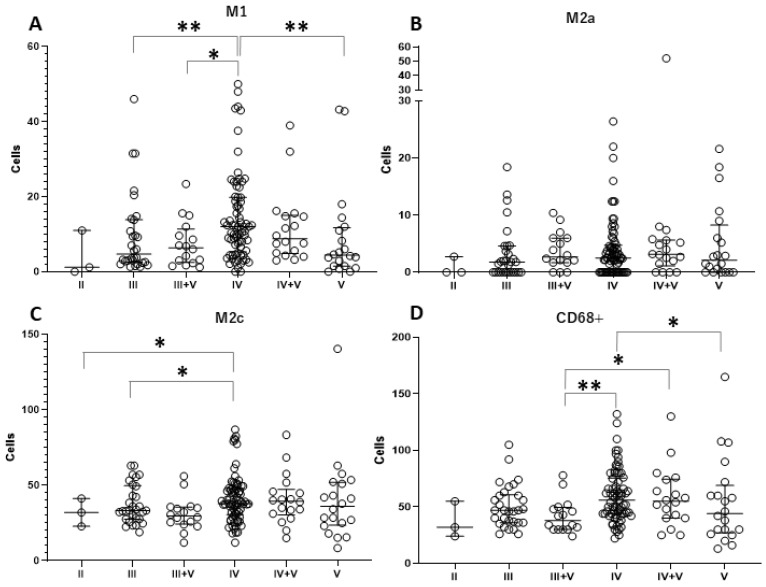
Subgroup analysis of the number of tubulointerstitial macrophage subgroups in different LN classes. Comparisons of tubulointerstitial macrophage subpopulations, including M1 macrophages (**A**), M2a macrophages (**B**), M2c macrophages (**C**), and CD68 + cells as total macrophages (**D**), in each LN class were performed by the Mann–Whitney U test. Dot plots represent individual numbers of macrophages (cells/high power field) in each category. The horizontal lines of each graphic are the median and interquartile range. Statistical significance was indicated by the values * *p* < 0.05 and ** *p* < 0.01.

**Figure 4:**
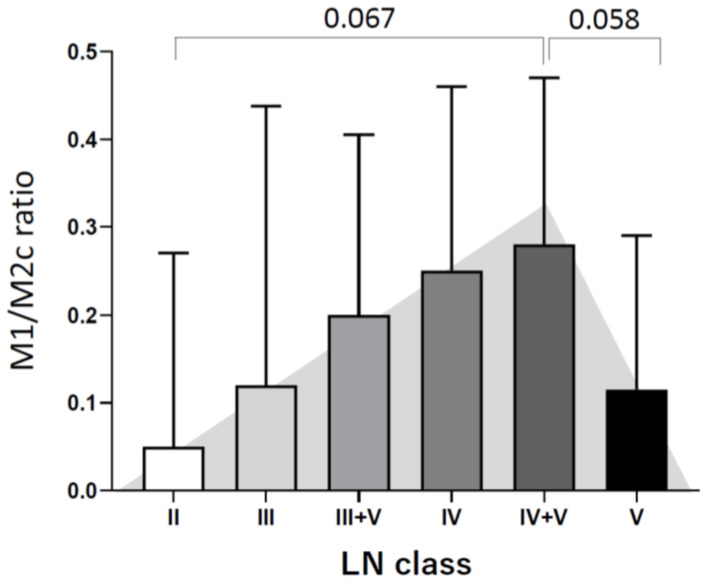
M1/M2c ratios in different LN classes. The plot shows the median and interquartile range of the M1/M2c ratio. No statistically significant difference was detected in this model. However, there was a tendency for the M1/M2c ratio to be higher in higher LN classes and much lower in LN class V. The comparisons between various LN classes were performed by the Mann–Whitney U test.

To compare the number of macrophage subpopulations, the Kruskal–Wallis test was initially tested among the various LN classes. We found that the numbers of M1 macrophage and total macrophage populations were significantly different among the LN classes, where *p* = 0.003 and 0.009, respectively (Table 2). The overall number of those macrophage subpopulations in each LN class versus other LN classes was visualized by histograms (Figure 2). Furthermore, post hoc analysis using the Mann–Whitney U test was used to investigate the number of macrophage subpopulations in the LN subclasses. This model demonstrated that the number of M1 macrophages in LN class IV was significantly higher than in classes III, III-V, and V (*p* = 0.009, 0.012, and 0.007, respectively) (Figure 3A). However, the M2a population was not different among LN classes (Figure 3B), while the M2c population in LN class IV was higher than in classes II and III, where *p* = 0.040 and 0.016, respectively (Figure 3C). In addition, the total number of macrophages (CD68+) in patients with LN classes III + V was significantly lower than in those with LN classes IV and IV + V (*p* = 0.002 and 0.040, respectively), while the number of total macrophages in LN class was significantly lower than in LN class IV (Figure 3D). The actual *p*-values of the subgroup analysis are given in Table 3. Furthermore, the M1/M2c ratio was investigated among the LN classes. The ratio was most likely to show a tendency to rise as severity increased, as indicated by a higher LN class. Unfortunately, no significant difference was observed in this ratio investigation (Figure 4).

### 3.3. Tubulointerstitial Macrophages Are Associated with High Activity and Chronicity Indices

In this study, we found that tubulointerstitial macrophage subpopulations and activity and chronicity indices were significantly correlated (Table 4). There was a highly significant correlation between the activity index and the number of M1 macrophages (*p* =< 0.001; rs = 0.41). In addition, there were strongly significant correlations between the chronicity index and the number of M1 macrophages (*p* =< 0.001; rs = 0.44), M2a macrophages (*p* =< 0.001; rs = 0.48), and total macrophages (*p* =< 0.001; rs = 0.42). Moreover, the number of M2c macrophages was observed to be correlated with the chronicity index as well (*p* = 0.024; rs = 0.18). Interstitial inflammation was noted to be correlated with the number of tubulointerstitial macrophage subpopulations, including M1 macrophages (*p* =< 0.001; rs = 0.43), M2a macrophages (*p*=< 0.001; rs = 0.41), M2c macrophages (*p* = 0.004; rs = 0.23), and total macrophages (*p* =< 0.001; rs = 0.44).

### 3.4. Tubulointerstitial Macrophages Are Associated with Decreased Renal Function

The relationship between tubulointerstitial macrophage subpopulations and renal function was determined by serum creatinine before and after 1-year treatment as well as 24 h urine protein. The correlations between serum creatinine before treatment and the number of M1 macrophages (*p* < 0.001; rs = 0.27), M2c macrophages (*p* < 0.001; rs = 0.28), and total macrophages (*p* < 0.001; 0.34) were observed to be significant. Furthermore, there was a strongly significant correlation between serum creatinine after 1-year treatment and M1 macrophages (*p* < 0.001; rs = 0.27) and total macrophages (*p* < 0.001; 0.30). In contrast, no correlation was found between the tubulointerstitial macrophage subpopulation and 24 h urine protein (Table 5).

## 4. Discussion

SLE is a chronic systemic autoimmune disease with diverse clinical manifestations characterized by immune system infiltration and inflammation in damaged organs covering the skin, lungs, joints, kidneys, and central nervous system [2,4]. The abnormalities in the activation state of circulating and tissue macrophages in patients with SLE are crucial factors in the occurrence and development of this disease [11,12,13]. Depleting macrophages attenuates skin and kidney disease severity, which indicates the vital function of macrophages in SLE pathogenesis [20,21,22]. The pro-inflammatory patrolling monocytes that accumulate in the glomeruli in SLE patients and lupus mice are the main components of lupus glomerular or tubulointerstitial inflammation [22].

LN class III and class IV lesions determine the hypercellularity of glomerular cells [16,17] and whether the presence of inflammatory cells in the mesangium indicates a more active lesion. This study revealed a considerable correlation between tubulointerstitial macrophage subpopulations and the lupus nephritis classification. The number of M1 macrophages and the total number of macrophages were correlated with classes IV and IV + V lupus nephritis. Based on pathological characteristics, there was a greater correlation between the number of macrophages and LN class IV than class V. Class IV possessed the most severe characteristics. Due to M1 macrophages’ function in promoting inflammation, there was a direct correlation between the total number of macrophages and the proportion of macrophages that resembled M1 macrophages. Other tubulointerstitial macrophages, such as M2a and M2c macrophages, did not correlate with lupus nephritis classes. There was no correlation between the number of macrophage subpopulations and the other LN classes. However, it was noted that the population in the current study comprised primarily cases of LN class IV, with only a few other classes represented. This issue also necessitates additional research in the future.

The role of macrophages in lupus nephritis aligns with findings regarding other immunologic cell biomarkers involved in lupus flares. For example, as highlighted by recent research [23], biomarkers such as T-regulatory (Treg) cells and B-cell subsets, including plasmablasts, also significantly contribute to lupus activity and flares. These cells participate in modulating immune tolerance and autoantibody production, emphasizing the interconnected nature of immune cell dysregulation in SLE. Comparing macrophage polarization with other biomarkers, such as the Treg/Th17 balance, may provide a more comprehensive understanding of the immune environment driving lupus pathogenesis. Further studies integrating macrophage and lymphocyte biomarkers could refine predictive tools for lupus activity and therapeutic responses.

In our study, we found a significant correlation between the activity and chronicity indices and the number of M1-like macrophages. In addition, we observed a correlation between the numbers of M2a, M2c, and total macrophages and the chronicity index. Our results are similar to those previously published [23,24]. We also observed that the number of M1 macrophages was strongly correlated with the activity index. A high activity index will follow if high M1 cell numbers are observed. It is possible that M1 macrophages are key cells in triggering the inflammatory processes that occur in LN, causing tissue reactions in different forms that we can evaluate from different features in the activity index. M1 macrophages were present in high numbers, as demonstrated by the elevated M1 macrophage markers, in LN with a high activity index. In contrast, the M2a and M2c subpopulations were reduced in the same cases, as shown in previous studies [24,25,26,27], potentially contributing to an anti-inflammatory imbalance. Nevertheless, their rapid and robust response comes at the expense of accuracy. For instance, cytokines and reactive oxygen species produced by M1 macrophages can cause endothelial dysfunction and renal cell death. Furthermore, the prolonged infiltration of M1 macrophages and other inflammatory components leads to the deterioration of renal function, while M2 macrophages induce fibrosis.

There is evidence to suggest that the balance between M1 and M2 macrophages is important for maintaining renal function. For example, studies have shown that an increase in M1 macrophages and a decrease in M2 macrophages are associated with renal dysfunction in various kidney diseases, such as diabetic nephropathy, lupus nephritis, and ischemia–reperfusion injury [24,27]. M1 macrophages are thought to play a pro-inflammatory role by producing cytokines that promote inflammation and fibrosis, while M2 macrophages are thought to play an anti-inflammatory role by producing cytokines that promote tissue repair and remodeling. In lupus nephritis, the accumulation of M1 macrophages in the kidneys is thought to contribute to renal injury and inflammation.

We also found that there was a strong correlation between the numbers of M1 and serum creatinine levels, both before and after treatment, where higher serum creatinine levels indicated decreased kidney function. It is possible that, in addition to having a significant role in stimulating inflammation, M1 macrophages also have a direct relationship with this type of cell volume, which causes more severe tissue inflammation. This is shown by where renal function decreases and the amount of damage that occurs. While this study focused on the association between macrophage polarization and lupus nephritis indices, integrating data on serum biomarkers, such as anti-double-strand DNA (anti-dsDNA) antibodies and complement (C3 and C4) levels, could provide a more comprehensive understanding of the disease activity and its correlation with macrophage subpopulations. These biomarkers are routinely used to monitor lupus activity and could serve as valuable complementary tools in assessing disease mechanisms and progression. Future studies incorporating these parameters alongside tissue-based findings would enhance the robustness of LN pathophysiology insights.

Overall, the balance between M1 and M2 macrophages appears to be critical for maintaining renal function and preventing renal injury and dysfunction. Further research is needed to fully understand the mechanisms by which macrophage subpopulations contribute to renal function and to develop novel therapeutic strategies targeting these cells in kidney diseases.

### Limitations of This Study

Our study had a few limitations. Renal biopsies on human samples can only provide a snapshot of the disease. As a consequence, we know nothing about the dynamics of macrophage subtypes during the onset and progression of the disease, nor do we clearly understand the correlation between macrophage subtypes and kidney injury or renal function. Additional experimental studies, such as animal or in vitro research, are necessary to clarify these issues. Additionally, we were unable to conduct a comprehensive analysis of other clinical symptoms due to the fact that the classification criteria for SLE, including the SLEDAI score, 2012 SLICC, and 2016 ACR/EULAR, are varied among clinicians. We will endeavor to analyze them all in accordance with the same classification criteria for SLE in our subsequent work and attempt to establish a connection with the information we have gathered.

## 5. Conclusions

In lupus nephritis, the majority of macrophages are M2 macrophages, which play a crucial role in the chronicity of this disease. M1 macrophages were found to be predominantly associated with inflammation and higher activity indices, indicating their involvement in acute disease processes. In contrast, M2 macrophage subpopulations correlated more with chronicity indices, suggesting their role in the progression and fibrosis of renal tissues. The findings highlight the distinct roles of M1 and M2 macrophages in lupus nephritis pathogenesis and suggest that macrophage polarization profiles could serve as potential biomarkers and therapeutic targets for managing this disease. Further studies are needed to explore the dynamic balance between these macrophage subtypes and their implications for disease outcomes.

## Figures and Tables

**Table 1:** Demographic and clinical characteristics of patients with lupus nephritis.

Variable	
**Age (years), mean ± SD**	33.14 ± 13.35
**Sex, n (%)**	
**Male**	141 (88.1)
**Female**	19 (11.9)
**ISN/RPS classification, n (%)**	
**Class II**	3 (1.9)
**Class III**	30 (18.8)
**Class III + V**	16 (10.0)
**Class IV**	73 (45.6)
**Class IV + V**	18 (11.3)
**Class V**	20 (12.5)
**Laboratory data, median (IQR)**	
**Serum creatinine before treatment**	1.18 (0.75–1.86)
**Serum creatinine after 1-year treatment**	0.7 (0.55–0.88)
**24 h urine protein before treatment**	1071 (621–1917)
**Urine protein dipstick test (before treatment), n (%)**	
**1+**	10 (6.3)
**2+**	29 (18.1)
**3+**	46 (28.7)
**4+**	75 (46.9)

**Table 2:** Comparison of tubulointerstitial macrophage subpopulations by LN classification.

Macrophage Sub-Populations	LN Classification; Median (IQR)						*p*-Value
	II	III	III + V	IV	IV + V	V	
**M1** **(iNOS+)**	1 (0.6–6.1)	4.7 (2.6–13.8)	6.4 (2.7–10.8)	12 (5.6–19.8)	9 (5–14.8)	4 (1.5–11.5)	0.003 *
**M2a** **(CD206+)**	0 (0–1.38)	1.8 (0–4.6)	2.7 (1.7–6)	2.5 (0–4.6)	3 (1.5–5.2)	2 (0–7.5)	0.53
**M2c** **(CD163+)**	32 (27.4–36.6)	33.3 (27.6–49.4)	29.7 (24.8–35.4)	37.8 (31.2–48)	40 (31.2–45.8)	34 (24–47.1)	0.13
**Macrophage** **(CD68+)**	32 (28–43.5)	47 (36–60)	38 (30–49)	56 (44–75)	55 (40–74)	42 (28–60)	0.009 *

* *p* < 0.05 was considered statistically significant, as provided by the Kruskal–Wallis test.

**Table 3:** *p*-values from a post hoc subgroup analysis of the number of tubulointerstitial macrophage subgroups in different LN classes.

Subgroup Comparison	M1 (iNOS+)	M2a (CD206+)	M2c (CD163+)	Total Macrophages (CD68+)
**II vs. III**	0.150	0.397	0.153	0.178
**II vs. III + V**	0.198	0.142	0.113	0.653
**II vs. IV**	0.064	0.189	0.040 *	0.074
**II vs. IV + V**	0.088	0.148	0.069	0.131
**II vs. V**	0.337	0.208	0.182	0.565
**III vs. III + V**	0.936	0.173	0.364	0.111
**III vs. IV**	0.009 *	0.206	0.016 *	0.053
**III vs. IV + V**	0.064	0.214	0.135	0.263
**III vs. V**	0.607	0.435	0.936	0.388
**III + V vs. IV**	0.012 *	0.697	0.328	0.002 *
**III + V vs. IV + V**	0.105	0.800	0.639	0.040 *
**III + V vs. V**	0.556	0.665	0.432	0.868
**IV vs. IV + V**	0.417	0.778	0.677	0.679
**IV vs. V**	0.007 *	1.000	0.051	0.037 *

Post hoc analysis was analyzed by the Mann–Whitney U test in order to compare each LN class group. * *p*-value ≤ 0.05 was considered statistically significant.

**Table 4:** Correlations between tubulointerstitial macrophage subpopulations and activity index and chronicity index.

Variable		Macrophage Subpopulation, n (%)			
		M1 (iNOS+)	M2a (CD206+)	M2c (CD163+)	Total Macrophages (CD68+)
**Activity index**					
	Spearman’s rho ^a^	0.41 **	0.12	0.07	0.22 *
	*p*-value	<0.001	0.13	0.40	0.005
**Chronicity index**					
	Spearman’s rho ^a^	0.44 **	0.48 **	0.18 *	0.42 **
	*p*-value	<0.001	<0.001	0.024	<0.001
**Interstitial inflammation**					
	Spearman’s rho ^a^	0.43 **	0.41 **	0.23 *	0.44 **
	*p*-value	<0.001	<0.001	0.004	<0.001

Abbreviations: Spearman’s rho, Spearman’s rank correlation coefficient. * Correlation was significant at *p* < 0.05. ** Correlation was highly significant at *p* < 0.01. ^a^ Correlation coefficient estimated by Spearman’s rank correlation.

**Table 5:** Correlations between tubulointerstitial macrophage subpopulations and renal function.

Variable		Macrophage Subpopulation, n (%)			
		M1 (iNOS+)	M2a (CD206+)	M2c (CD163+)	Total Macrophages (CD68+)
**Serum creatinine before treatment**					
	Spearman’s rho ^a^	0.27 *	0.17 *	0.28 **	0.34 *
	*p*-value	<0.001	0.036	<0.001	<0.001
**Serum creatinine after 1-year treatment**					
	Spearman’s rho ^a^	0.27 **	0.15	0.22 *	0.30 **
	*p*-value	<0.001	0.063	0.007	<0.001
**24 h urine protein before treatment**					
	Spearman’s rho ^a^	0.14	0.09	0.13	0.17 *
	*p*-value	0.082	0.287	0.115	0.04

Abbreviations: Spearman’s rho, Spearman’s rank correlation coefficient. * Correlation was significant at *p* < 0.05. ** Correlation was highly significant at *p* < 0.01. ^a^ Correlation coefficient estimated by Spearman’s rank correlation.

## Data Availability

All data presented in this study and are available on request from the corresponding author.

## References

[1] Mohan C., Putterman C. (2015). Genetics and pathogenesis of systemic lupus erythematosus and lupus nephritis. Nat. Rev. Nephrol..

[2] Jakes R.W., Bae S.C., Louthrenoo W., Mok C.C., Navarra S.V., Kwon N. (2012). Systematic review of the epidemiology of systemic lupus erythematosus in the Asia-Pacific region: Prevalence, incidence, clinical features, and mortality. Arthritis Care Res..

[3] Benjakul N., Henriksen K.J., Chang A. (2024). Non-neoplastic Kidney Diseases in Adult Tumor Nephrectomy and Nephroureterectomy Specimens in a Southeast Asian Tertiary Medical Center. Vajira Med. J. J. Urban Med..

[4] Kasitanon N., Louthrenoo W., Sukitawut W., Vichainun R. (2002). Causes of death and prognostic factors in Thai patients with systemic lupus erythematosus. Asian Pac. J. Allergy Immunol..

[5] Davidson A., Aranow C. (2006). Pathogenesis and treatment of systemic lupus erythematosus nephritis. Curr. Opin. Intern. Med..

[6] Triantafyllopoulou A., Franzke C.W., Seshan S.V., Perino G., Kalliolias G.D., Ramanujam M., Van Rooijen N., Davidson A., Ivashkiv L.B. (2010). Proliferative lesions and metalloproteinase activity in murine lupus nephritis mediated by type I interferons and macrophages. Proc. Natl. Acad. Sci. USA.

[7] Sahu R., Bethunaickan R., Singh S., Davidson A. (2014). Structure and Function of Renal Macrophages and Dendritic Cells from Lupus-Prone Mice. Arthritis Rheumatol..

[8] Tugal D., Liao X., Jain M.K. (2013). Transcriptional Control of Macrophage Polarization. Arterioscler. Thromb. Vasc. Biol..

[9] Martinez F.O., Sica A., Mantovani A., Locati M. (2008). Macrophage activation and polarization. Front. Biosci..

[10] Schwartz Y.S., Svistelnik A.V. (2012). Functional phenotypes of macrophages and the M1–M2 polarization concept. Part I. Proinflammatory phenotype. Biochemistry.

[11] Guo S., Wietecha T.A., Hudkins K.L., Kida Y., Spencer M.W., Pichaiwong W., Kojima I., Duffield J.S., Alpers C.E. (2011). Macrophages are essential contributors to kidney injury in murine cryoglobulinemic membranoproliferative glomerulonephritis. Kidney Int..

[12] Anders H.-J., Ryu M. (2011). Renal microenvironments and macrophage phenotypes determine progression or resolution of renal inflammation and fibrosis. Kidney Int..

[13] Arnold C.E., Whyte C.S., Gordon P., Barker R.N., Rees A.J., Wilson H.M. (2013). A critical role for suppressor of cytokine signalling 3 in promoting M1 macrophage activation and function in vitro and in vivo. Immunology.

[14] Lee S., Huen S., Nishio H., Nishio S., Lee H.K., Choi B.-S., Ruhrberg C., Cantley L.G. (2011). Distinct Macrophage Phenotypes Contribute to Kidney Injury and Repair. J. Am. Soc. Nephrol..

[15] Filardy A.A., Pires D.R., Nunes M.P., Takiya C.M., Freire-De-Lima C.G., Ribeiro-Gomes F.L., DosReis G.A. (2010). Proinflammatory Clearance of Apoptotic Neutrophils Induces an IL-12lowIL-10high Regulatory Phenotype in Macrophages. J. Immunol..

[16] Weening J.J., D’agati V.D., Schwartz M.M., Seshan S.V., Alpers C.E., Appel G.B., Balow J.E., Bruijn J.A.N.A., Cook T., Ferrario F. (2004). The classification of glomerulonephritis in systemic lupus erythematosus revisited. Kidney Int..

[17] Bajema I.M., Wilhelmus S., Alpers C.E., Bruijn J.A., Colvin R.B., Cook H.T., D’agati V.D., Ferrario F., Haas M., Jennette J.C. (2018). Revision of the International Society of Nephrology/Renal Pathology Society classification for lupus nephritis: Clarification of definitions, and modified National Institutes of Health activity and chronicity indices. Kidney Int..

[18] Roufosse C., Simmonds N., Clahsen-van Groningen M., Haas M., Henriksen K.J., Horsfield C., Loupy A., Mengel M., Perkowska-Ptasińska A., Rabant M. (2018). A 2018 Reference Guide to the Banff Classification of Renal Allograft Pathology. Transplantation.

[19] Nonsiri P., Srivilai P., Onkaew S., Benjakul N. (2023). The effectiveness of using dye models for small tissue biopsies in the surgical pathology laboratory. Folia Histochem. Cytobiol..

[20] Ma W.T., Gao F., Gu K., Chen D.K. (2019). The Role of Monocytes and Macrophages in Autoimmune Diseases: A Comprehensive Review. Front. Immunol..

[21] Chalmers S.A., Chitu V., Herlitz L.C., Sahu R., Stanley E.R., Putterman C. (2015). Macrophage depletion ameliorates nephritis induced by pathogenic antibodies. J. Autoimmun..

[22] Kuriakose J., Redecke V., Guy C., Zhou J., Wu R., Ippagunta S.K., Tillman H., Walker P.D., Vogel P., Häcker H. (2019). Patrolling monocytes promote the pathogenesis of early lupus-like glomerulonephritis. J. Clin. Investig..

[23] Kosałka-Węgiel J., Jakieła B., Dziedzic R., Milewski M., Siwiec-Koźlik A., Zaręba L., Bazan-Socha S., Sanak M., Musiał J., Korkosz M. (2024). Circulating B Lymphocyte Subsets in Patients with Systemic Lupus Erythematosus. Medicina.

[24] Li J., Liu C.-H., Xu D.-L., Gao B. (2015). Significance of CD163-Positive Macrophages in Proliferative Glomerulonephritis. Am. J. Med Sci..

[25] Barros M.H.M., Hauck F., Dreyer J.H., Kempkes B., Niedobitek G. (2013). Macrophage Polarisation: An Immunohistochemical Approach for Identifying M1 and M2 Macrophages. PLoS ONE.

[26] Ohlsson S.M., Linge C.P., Gullstrand B., Lood C., Johansson Å., Ohlsson S., Lundqvist A., Bengtsson A.A., Carlsson F., Hellmark T. (2014). Serum from patients with systemic vasculitis induces alternatively activated macrophage M2c polarization. Clin. Immunol..

[27] Olmes G., Büttner-Herold M., Ferrazzi F., Distel L., Amann K., Daniel C. (2016). CD163+ M2c-like macrophages predominate in renal biopsies from patients with lupus nephritis. Arthritis Res. Ther..

